# Connectome-based predictive modeling of Internet addiction symptomatology

**DOI:** 10.1093/scan/nsae007

**Published:** 2024-02-09

**Authors:** Qiuyang Feng, Zhiting Ren, Dongtao Wei, Cheng Liu, Xueyang Wang, Xianrui Li, Bijie Tie, Shuang Tang, Jiang Qiu

**Affiliations:** Center for Studies of Education and Psychology of Ethnic Minorities in Southwest China, Southwest University (SWU), Chongqing 400715, China; Key Laboratory of Cognition and Personality (SWU), Ministry of Education, Chongqing 400715, China; Key Laboratory of Cognition and Personality (SWU), Ministry of Education, Chongqing 400715, China; School of Psychology, Southwest University (SWU), Chongqing 400715, China; Key Laboratory of Cognition and Personality (SWU), Ministry of Education, Chongqing 400715, China; School of Psychology, Southwest University (SWU), Chongqing 400715, China; Key Laboratory of Cognition and Personality (SWU), Ministry of Education, Chongqing 400715, China; School of Psychology, Southwest University (SWU), Chongqing 400715, China; Key Laboratory of Cognition and Personality (SWU), Ministry of Education, Chongqing 400715, China; School of Psychology, Southwest University (SWU), Chongqing 400715, China; Key Laboratory of Cognition and Personality (SWU), Ministry of Education, Chongqing 400715, China; School of Psychology, Southwest University (SWU), Chongqing 400715, China; Center for Studies of Education and Psychology of Ethnic Minorities in Southwest China, Southwest University (SWU), Chongqing 400715, China; Key Laboratory of Cognition and Personality (SWU), Ministry of Education, Chongqing 400715, China; Key Laboratory of Cognition and Personality (SWU), Ministry of Education, Chongqing 400715, China; School of Psychology, Southwest University (SWU), Chongqing 400715, China; Key Laboratory of Cognition and Personality (SWU), Ministry of Education, Chongqing 400715, China; School of Psychology, Southwest University (SWU), Chongqing 400715, China; Southwest University Branch, Collaborative Innovation Center of Assessment Toward Basic Education Quality, Beijing Normal University, Beijing 100000, China

**Keywords:** Internet addiction symptomatology, connectome-based predictive modeling, resting-state functional connectivity

## Abstract

Internet addiction symptomatology (IAS) is characterized by persistent and involuntary patterns of compulsive Internet use, leading to significant impairments in both physical and mental well-being. Here, a connectome-based predictive modeling approach was applied to decode IAS from whole-brain resting-state functional connectivity in healthy population. The findings showed that IAS could be predicted by the functional connectivity between prefrontal cortex with the cerebellum and limbic lobe and connections of the occipital lobe with the limbic lobe and insula lobe. The identified edges associated with IAS exhibit generalizability in predicting IAS within an independent sample. Furthermore, we found that the unique contributing network, which predicted IAS in contrast to the prediction networks of alcohol use disorder symptomatology (the range of symptoms and behaviors associated with alcohol use disorder), prominently comprised connections involving the occipital lobe and other lobes. The current data-driven approach provides the first evidence of the predictive brain features of IAS based on the organization of intrinsic brain networks, thus advancing our understanding of the neurobiological basis of Internet addiction disorder (IAD) susceptibility, and may have implications for the timely intervention of people potentially at risk of IAD.

## Introduction

In the last few decades, the Internet has become an indispensable part of human life. On the one hand, the Internet has brought convenience to human life and efficient work; while on the other hand, due to the increased dependence on the Internet, there is a consequent risk of addictive behavior (Chou *et al*., 2005; Alimoradi *et al*., 2019). Internet addiction symptomatology (IAS) refers to an individual’s loss of control over Internet use, leading to serious negative consequences, such as psychological problems, tense human relationships, sleeplessness and suicidal impulse (Akin and İSkender, 2011; Chi *et al*., 2019). Further insight into the neurocognitive underpinnings of IAS could provide information facilitating the prevention and treatment of Internet addiction disorder (IAD).

Recent neuroscientific research has demonstrated potential associations between IA and the functional and structural changes in the brain networks of affected individuals, which are critical for emotion regulation, inhibitory control and reward processing (Choi, 2017; Sharifat *et al*., 2018; Patil *et al*., 2021). For instance, those with IAS exhibited decreased activity and altered resting-state functional connectivity (rsFC) of several areas in the cognitive control network, such as the dorsolateral prefrontal cortex (DLPFC) and the anterior cingulate cortex (ACC) (Li *et al*., 2015; Dieter *et al*., 2017; Wang *et al*., 2017), both of which are functionally associated with inhibitory control and emotion processing (Li *et al*., 2015; Patil *et al*., 2021). Furthermore, similar results have been found in structural studies, and a decreased gray matter volume in the DLPFC and ACC was observed in IAS, which possibly accounted for the reduced inhibitory control (Solly *et al*., 2022). Moreover, impaired inhibitory control is believed to be responsible for the development and maintenance of IAS (Dong *et al*., 2012a; Choi *et al*., 2014). At the large-scale network level, IAS is associated with imbalanced interactions among the default mode network, fronto-parietal network and salience network (Wang *et al*., 2017). In addition, an activity change in the reward system (e.g. the striatum) is an important feature of addictive behaviors (including IAS) (Koob and Le Moal, 2008; Dong *et al*., 2011; Koob and Volkow, 2016). Taken together, previous neuroimaging evidence indicates that IAS has an effect on the distribution networks at the system level of the brain, not just on isolated regions. Meanwhile, previous studies have mostly focused on individuals who met the diagnostic criteria for IAS, also known as IAD, and the results were produced by comparing patients with IAD and healthy control groups (Patil *et al*., 2021). Excessive use of the Internet can increase the propensity for IAS and the risk of developing IAD. Individuals with a heightened susceptibility to IAS may serve as a transitional group between healthy adults and individuals who have progressed to full-blown addiction. Clearly, healthy individuals who score higher on the IAS test may be more susceptible to IAD (Li *et al*., 2015); therefore, it is of great importance to investigate the neural mechanisms underlying individual differences in healthy subjects with a predisposition to IAS.

Recently, connectome-based predictive modeling (CPM) has emerged to link brain features and individual behavior (Shen *et al*., 2017). CPM is a novel data-driven method for constructing predictive models of individual behavior from functional connectivity during the resting state, allowing for a more accurate detection of individual variability (Shen *et al*., 2017). Previous studies have confirmed that this approach can be successfully used to predict individual personality traits and functional cognition (Yoo *et al*., 2018; Ren *et al*., 2021; Horien *et al*., 2022; Anderson and Barbey, 2023). In addition, CPM generally implements a built-in cross-validation procedure that includes estimating the model with training samples and testing the performance of the model with novel subjects. Therefore, predictive models enable the prediction of specific psychological behaviors, thus contributing to clinical practice and the need for physicians to make individualized assessments of symptom severity in order to find more targeted treatment options (Feng *et al*., 2019). Furthermore, predictive models have greater practical value than the commonly used group statistical methods because all available brain features are integrated into the predictive model with whole-brain analyses, in a way that contributes to enhanced statistical power and avoids multiple comparisons (Feng *et al*., 2018).

In light of the existing body of research, this study employed the CPM approach to investigate the neural predictive model for IAS. To ensure the specificity of the observed relationships between rsFC and IAS, several variables were controlled during the feature selection and model evaluation stages. A significant risk factor for addictive behaviors, namely negative emotion, was controlled based on prior findings (Santangelo *et al*., 2022). Additionally, gender, age and head motion parameters [mean framewise displacement (FD)] were included as control variables in our predictions, considering their documented associations with functional connectivity (Feng *et al*., 2018). Furthermore, to identify unique prediction networks specific to IAS, we aimed to assess the divergence between the sets of functional connections associated with IAS and alcohol use disorder symptomatology (AUDS, the range of symptoms and behaviors associated with alcohol use disorder). This comparative analysis sought to determine the degree to which the network of functional connections related to IAS differs from that of AUDS. In this way, we can also determine whether the CPM model can similarly predict tendencies toward a different addiction.

## Material and methods

### Participants

Two samples of participants were recruited for the present study. Both samples were recruited from Southwest University in Chongqing, China, and both completed the survey measures described below and underwent rsfMRI. It is important to note that all participants were right-handed, mentally healthy individuals with no history of mental illness. Written informed consent was obtained from each participant and they were compensated for their participation. The study protocol was approved by the Institutional Review Board of the Southwest University Brain Imaging Center.

Dataset 1 was obtained from a large sample dataset called the Gene Brain Behavior project (GBB). The project-related recruitment information and data presentations have been reported previously (Chen *et al*., 2019). A total of 689 participants from the GBB project were assessed for IAS and underwent resting-state scans. Of those who completed the IAS test, 512 participants completed state anxiety assessment, 671 participants completed depression assessment, 458 participants completed perceived stress assessment, 330 participants completed positive affect and negative affect assessment and 248 participants completed loneliness assessment. The missing values of behavior were supplemented by the random forest method. After the exclusion of subjects with excessive head movement (mean FD power > 0.3) during resting-state functional magnetic resonance imaging (fMRI), 677 participants were included in the subsequent analysis (mean age: 19.94 ± 7.13 years old; range: 16–25 years old; 191 males and 486 females).

To validate the predictive performance of the functional connectome on IAS, the Dataset 2 called the Southwest University Longitudinal Imaging Multimodal Project (SLIM) was used (Liu *et al*., 2017). A total of 115 participants completed resting-state scans and IAS measures (mean age: 21.89 ± 0.93 years old; range: 19–24 years old; 49 males and 66 females).

The AUDS data also come from SLIM. A total of 206 participants completed resting-state scans and AUDS measures, and excluded 1 participant with excessive head movement (mean FD power > 0.3). 205 participants were included in the subsequent analysis (mean age: 21.95 ± 1.01 years old; range: 19–25 years old; 89 males and 116 females). Of those who completed the AUDS test, 196 participants completed state anxiety assessment, 196 participants completed depression assessment, 199 participants completed perceived stress assessment, 196 participants completed positive affect and negative affect assessment and 199 participants completed loneliness assessment.

### Assessment of IAS

IAS was assessed using the Internet Addiction Tendency Scale in GBB, which was developed based on the definition that IAS is a type of psychological dependence on Internet use, including Internet-based relationship addiction, Internet-based entertainment addiction and Internet-based information collection addiction. The scale consists of 47 items and each item is scored on a 4-point Likert scale ranging from 1 (strongly disagree) to 4 (strongly agree). The higher scores on the scale indicate higher levels of IAS. The internal reliability of the scale in the original study was 0.87 (Chen and Huang, 2007).

IAS was assessed using the Internet Addiction Test (IAT) in SLIM. The IAT is a 20-item questionnaire that explores an individual’s Internet usage habits, their attitudes towards the Internet and the impact of Internet use on various aspects of their lives, such as compulsive use, withdrawal symptoms and related problems at school, work and sleep (Young, 2018). The validity and reliability of the IAT as a tool for assessing IAS have been confirmed (Widyanto *et al*., 2011). Each item in the questionnaire allows for a scaled selection ranging from 1 (‘not at all’) to 5 (‘always’), and higher total scores indicate a greater inclination towards addictive Internet usage. Individuals with higher IAT scores may be at a heightened risk of developing IAD.

### Negative emotion assessment

The negative emotion assessment included five questionnaires:

#### The Beck Depression Inventory (BDI)

The BDI is employed to assess the degree of depression among participants. This inventory consists of 21 questions that individuals respond to based on their experiences over the past week. Each question offers four answer options, arranged in a manner that reflects increasing severity of depressive symptoms (Beck, 1979).

#### The State Anxiety Inventory

The State Anxiety Inventory (SAI), developed by Spielberger in 1983, was utilized to evaluate the levels of individual’s anxious feelings (Spielberger, 2012). This inventory consists of 20 items, including sample statements such as ‘I am nervous’, ‘I feel upset’ and ‘I feel scared’. Participants were instructed to rate each item on a 4-point scale, reflecting the frequency of these feelings in general, ranging from ‘Almost Never’ to ‘Almost Always’. Higher scores on the inventory indicate elevated levels of anxiety.

#### Perceived stress

The Perceived Stress Scale (PSS) was utilized to evaluate the participants’ level of perceived stress in their daily life (Cohen *et al*., 1983). The PSS consisted of 10 self-assessment items, each rated on a 5-point Likert scale ranging from 0 to 4. The sum of these items yielded a stress scale score ranging from 0 to 40 points. A higher score on the stress scale indicated a higher level of perceived stress experienced by each individual in their daily life.

#### Positive and Negative Affect Scale

The Positive and Negative Affect Scale (PANAS) is a self-report questionnaire comprising 10 items to assess positive affect and another 10 items to measure negative affect (Watson *et al*., 1988). Each item is rated on a 5-point scale, ranging from 1 (not at all) to 5 (very much). For the purposes of this study, we focused solely on the total score of negative affect, referred to as PANAS_NA_Score.

#### University of California, Los Angeles Loneliness Scale

The University of California, Los Angeles (UCLA) Loneliness Scale (LS) is a self-report questionnaire consisting of 20 items, developed by Russell (Russell *et al*., 1980). Its purpose is to assess an individual’s subjective experience of loneliness and social isolation. Participants rate each statement on a 4-point Likert scale ranging from 1 (never) to 4 (often), indicating the frequency with which they relate to each statement. Higher scores on the scale indicate higher levels of loneliness, while lower scores indicate lower levels of loneliness.

Considering the multidimensional construct of negative emotion, the principal component method was used for exploratory factor analysis. The results showed that there was only one factor with an eigenroot greater than 1 (characteristic root = 2.87), and the total interpretation rate of variance was 72.12%. The factor loading of each index was as follows: state anxiety, 0.78; depression, 0.636; perceived stress, 0.786; negative affect, 0.818; and loneliness, 0.756. The results demonstrate that the five subindexes can be combined into one principal component, which is the comprehensive index of negative emotion. The comprehensive index of negative emotion was the sum of the products of the standard scores of the five subindexes and their corresponding factor loading.

### Assessment of AUDS

AUDS was evaluated using the Michigan Alcoholism Screening Test (MAST) (Selzer, 1971). The MAST is a comprehensive and standardized assessment tool specifically designed to measure the severity of alcohol dependence. It consists of 25 questions, each requiring a yes or no response. Each item is assigned a score of either 0, 1, 2 or 5 points, resulting in a total score ranging from 0 to 53. The total score is derived by summing the scores of all individual items. In this way, the MAST provides a reliable and quantifiable measure of alcohol dependence.

### MRI data acquisition

Functional and structural data were obtained using a Siemens 3 T Trio scanner (Siemens Medical Systems, Erlangen, Germany) at the Brain Imaging Center of Southwest University.

Resting-state fMRI data were obtained using a Gradient Echo-type Echo Planar Imaging (GRE-EPI) sequence: repetition time (TR) = 2000 ms, echo time (TE) = 30 ms, flip angle (FA) = 90^°^, field of view (FOV) = 220 × 220 mm^2^, slices = 32, thickness = 3 mm, interslice gap = 1 mm and voxel size = 3.4 × 3.4 × 4 mm^3^. High-resolution, three-dimensional T1-weighted structural images were obtained using a magnetization prepared rapid acquisition gradient-echo (MPRAGE) sequence: TR = 1900 ms, TE = 2.52 ms, FA = 9^°^, slices = 176, FOV = 256 × 256 mm^2^, thickness = 1 mm, and voxel size = 1 × 1 × 1 mm^3^.

### Preprocessing of MRI data

The resting-state fMRI data were preprocessed using the data processing assistant for resting-state fMRI software (http://www.restfmri.net/forum/DPARSF) (Yan and Zang, 2010). First, it takes time for the gradient field to stabilize and for the subject to adapt when he or she enters the MRI machine; thus, functional images were excluded from the first 10 time points and the remaining 232 images were included in the image analysis. Next, the usual preprocessing procedures, including correction of the slice timing, realignment, spatial normalization to the Montreal Neurological Institute (MNI) template and resampling, were carried out. Additionally, nuisance variables, including the global mean signal, white matter, cerebrospinal fluid and 24 motion parameters for head movement, were regressed out to the effect of the subject’s head movements and other brain tissue signals. All images were spatially normalized to the MNI template and resampled into 3 mm cubic voxels, followed by spatial smoothing with a full-width at half maximum of the Gaussian kernel of 4 mm. Finally, the smoothed data were filtered using a band-pass filter (0.01–0.1 Hz) to reduce the effect of physiological noise due to respiration and heartbeat.

### rsFC feature extraction

**Fig. 1.**
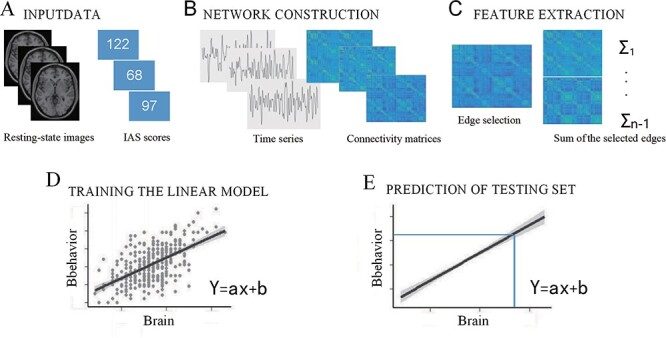
The schematic flow of the prediction method using patterns of resting-state brain connectivity.

In the current study (Figure 1), network nodes were defined by using a functional brain atlas that includes 268 nodes all over the brain, including the cerebellum and brainstem (Shen *et al*., 2010). The atlas was derived from a graph theory-based parcellation algorithm, which maximized the similarity of the voxelwise time series within each node. Compared to the nodes defined by the automatic anatomic labeling atlas, the 268-node atlas comprises nodes with a more coherent time series (Shen *et al*., 2013).

To construct the whole-brain rsFC matrices, the Graph Theoretical Network Analysis (GRETNA) toolbox was used to compute the time course of each node by averaging the blood oxygen level-dependent signal of all of its constituent voxels at each time point (Wang *et al*., 2015). The network edges were defined as the correlations (Pearson’s *r*) between time courses of each pair of nodes (i.e. functional connectivity between each pair of nodes). Fisher’s *r*-to-*z* transformation was then conducted to improve the normality of the correlation coefficients, resulting in a 268 × 268 symmetric connectivity matrix that represented the set of edges/connections in each participant’s resting-state connectivity profile.

### Connectome-based predictive modeling

After extracting the rsFC characteristics, the CPM approach was used to construct the neural predictive models for IAS. To evaluate the prediction performance, the leave-one-out cross-validation (LOOCV) method was employed, which allowed examining whether the rsFC strength can predict IAS for each individual. In each LOOCV iteration, data from *n* − 1 participants (the training set) were used to build a predictive model, which was then used to predict the scores of the remaining participants (the testing set). For the training dataset, the IAS scores and the edges in the connectivity matrix were normalized. Next, Pearson’s correlation coefficients between the IAS scores and each edge in the rsFC matrix were calculated. The edges that were significantly correlated with the IAS scores (*P* < 0.01) were selected and divided into positive (i.e. positively related to the IAS scores) or negative (i.e. negatively related to the IAS scores) networks (Rosenberg *et al*., 2016). The rsFC strength was then calculated by summing all selected edges in a positive or negative network. Finally, linear regression models that fit positive and negative relationships between the IAS scores and rsFC strength, respectively, were estimated. During the testing procedure, each participant’s positive and negative network strengths were normalized using the parameters acquired during the training procedure, and then the trained models were used to predict the participant’s IAS score in the testing set.

After LOOCV, the performance of the predictive models was evaluated by Pearson’s correlation coefficient (*r*) between the actual IAS scores and the predicted IAS scores for the positive and negative models, respectively. Permutation tests were used to evaluate the significance of the positive and negative predictive models. In each iterations, IAS scores were assigned randomly to different subjects as a way to shuffle the true brain-behavior mapping. Then, the same procedure for estimating the predictive models was applied to compute the *r*-values between the actual and predicted rating scores. The null hypothesis distributions of the *r*-values for the positive and negative models formed after completing 1000 permutations were determined. The *P*-value was the proportion of the permutation-generated *r*-values greater than the true predictive *r*-values. The significance level of the permutation test was set at 0.05.

### Control analyses

To ensure the specificity of the observed associations between rsFC and IAS, several variables were controlled for in this study. First, considering that negative emotion has been identified as a significant risk factor for addictive behaviors (Santangelo *et al*., 2022), we included negative emotion as a control variable. Additionally, gender, age and mean FD were controlled for, as these factors have also been found to be related to functional connectivity (Feng *et al*., 2018). In these analyses, new predictive networks were constructed by employing those edges whose partial Pearson correlation with IAS scores while controlling for confounding variables passed the *P* < 0.01 threshold (see also Shen *et al*., 2017). The performance of the prediction model was assessed using the partial Pearson correlation coefficient (*r*) between actual IAS scores and predicted IAS scores after controlling for confounding variables.

### Contributing network in the prediction of IAS scores

Each LOOCV iteration selected a slightly different edge in the rsFC matrix. To characterize the neural substrates of the contributing network, the network was defined as the set of edges that were present in every iteration of the LOOCV described earlier. Afterwards, the 268 nodes were grouped into 10 macroscale brain regions, including the prefrontal cortex (46 nodes), motor lobe (21 nodes), insular lobe (7 nodes), parietal lobe (27 nodes), temporal lobe (39 nodes), occipital lobe (25 nodes), limbic lobe (36 nodes), cerebellum (41 nodes), subcortical lobe (17 nodes) and brainstem (9 nodes) (Shen *et al*., 2010). The number of edges between each pair of macroscale regions was then calculated. In addition, to generate a simple interpretation of the importance of each node within the contributing network, the node strength was calculated (He *et al*., 2021). The node strength is the sum of the weights of links connected to a node and is a commonly used metric to weigh the importance of each node in the contributing network.

### Validation analysis with different cross-validation schemes

The main results were further validated with a 10-fold cross-validation. All of the participants were divided into 10 subsets, nine subsets of which served as the training set (90% of participants) and the remaining subset served as the testing set (10% of participants). The training set was normalized and used to train a linear predictive model, which then was used to predict the scores of the normalized testing data. The parameters acquired from the training data were used to normalize the testing data. This procedure was repeated 10 times so that each subset was used as the testing set once. Finally, the *r*-values between the true and predicted scores were calculated across all participants. Since the entire dataset was randomly divided into 10 subsets, the prediction performance might depend on the division of data. To address this issue, the 10-fold cross-validation was repeated 100 times and the results were averaged to produce a final prediction performance. A 1000 times permutation test was then applied to test the significance of the prediction performance.

### External generalizability

We examined the external predictive efficacy of the IAS-related edges identified in the discovery sample by assessing their significance in predicting IAS within an independent validation sample of 115 participants (Dataset 2). The evaluation of results across different samples provides a robust method to determine the generalizability of CPM-based findings (Shen *et al*., 2017). To accomplish this, we calculated the summed negative IAS network strengths for each participant in the validation sample. These strengths were obtained by aggregating the relevant edges identified through the CPM process in Dataset 1. Subsequently, these network strengths were inputted into regression models to generate predicted IAS values, similar to the approach used in Dataset 1. The regression model parameters (slopes and intercepts) employed to generate predicted IAS values in Dataset 2 were derived from leave-one-out iterations performed on Dataset 1 to generate predicted IAS values for each subject left out. In Dataset 1, a total of 677 iterations of each model type were executed to generate predicted IAS values for a distinct left-out subject within that sample. The average of these model parameters was utilized to construct the regression models for predicting IAS values in Dataset 2.

To evaluate the effectiveness of these predictions, similar to Dataset 1, we established a correlation between the predicted IAS scores and the observed IAS scores. If the predicted IAS scores, generated solely using information from Dataset 1, exhibit a significant relationship with the observed IAS scores in the external validation dataset, it would offer robust evidence supporting the generalizability of our findings.

### Comparing the sets of functional connections related to IAS to those related to AUDS

Finally, to identify unique prediction networks specific to IAS, we set out to assess the extent to which the set of functional connections related to IAS different from the set of functional connections related to AUDS. To do this, we constructed the neural predictive models of AUDS, comparing the sets of functional connections related to IAS to those related to AUDS.

## Results

### Behavioral performance

Table 1 describes the descriptive statistics of behavioral measurements in Dataset 1. For details on Dataset 2, please see Supplementary Table S1.

**Table 1.** The descriptive statistics of behavioral measurements in Dataset 1

Outcomes	*N*	Mean	SD	Range	Cronbach’s alpha coefficients
IAS	677	101.07	20.41	46–167	0.86
Depression	671	6.93	6.35	0–40	0.86
State anxiety	512	40.39	9.30	20–72	0.92
Perceived stress	458	17.07	4.72	0–33	0.75
Negative affect	330	19.57	5.72	10–46	0.87
Loneliness	248	41.76	7.48	21–62	0.86

### Prediction analysis using cross-validation

**Fig. 2.**
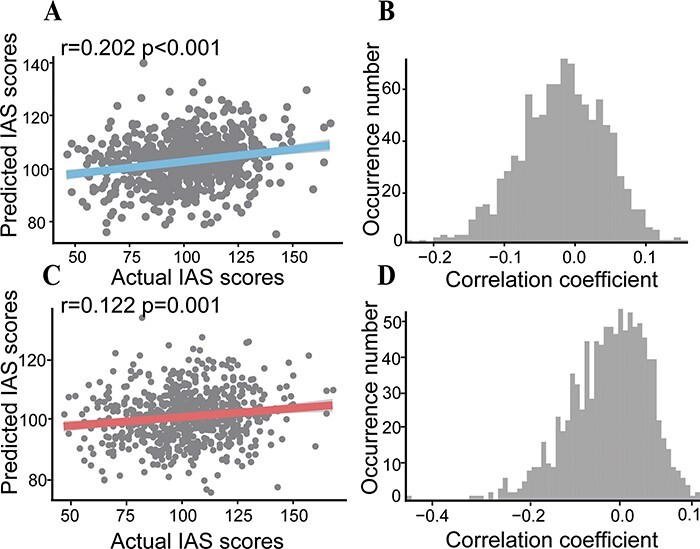
The performance of the predictive model. (A) The correlation between the actual and predicted IAS scores. (B) The permutation distribution of the correlation coefficient (*r*) for the prediction analysis. (C) The correlation between the actual and predicted IAS scores after controlling for head motion, age, gender and negative emotion scores. (D) The permutation distribution of the correlation coefficient (*r*) for the prediction analysis after controlling for head motion, age, gender and negative emotion scores.

Based on the construction of the positive and negative predictive models related to IAS, it was found that the brain features (i.e. rsFC) in the negative network could successfully be used to predict the IAS scores in the independent subjects (correlation between the actual and predicted scores: *r* = 0.202, *P*_perm_ = 0.001, permutation test, Figure 2). What is inconsistent with our hypothesis is that the functional connectivity in the positive network could not reliably the predict IAS scores (correlation between the actual and predicted scores: *r* = − 0.06, *P* > 0.05). After controlling for head motion, age, gender, negative emotion scores, negative networks still significantly predicted IAS scores (correlation between the actual and predicted scores: *r*_partial_ = 0.122, *P*_perm_ = 0.035, Figure 2).

### Contributing networks in the prediction of IAS scores

**Fig. 3.**
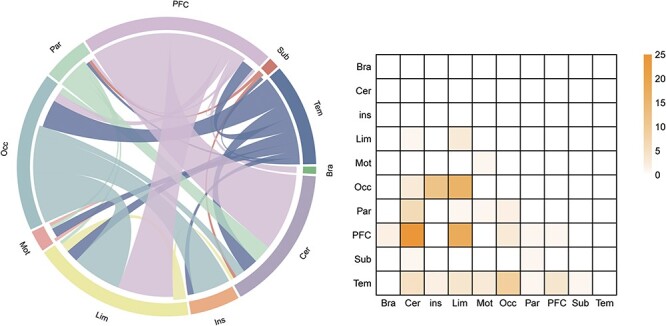
Functional connections predicting IAS scores after controlling for head motion, age, gender and negative emotion scores. The left graph shows the connections within and between each macroscale regions. The right graph depicts the connections plotted as number of edges within and between each pair of macroscale regions. PFC, prefrontal cortex; Mot, motor lobe; Ins, insular lobe; Par, parietal lobe; Tem, temporal lobe; Occ, occipital lobe; Lim, limbic lobe; Cer, cerebellum; Sub, subcortical lobe; Bsm, brainstem.

In all iterations of LOOCV, the number of predictive model edges varied from 392 to 478, with 127 edges appearing in each iteration defined as the contributing network after controlling for confounding variables (head motion, age, gender, negative emotion scores). Based on the macroscale regions, the contributing network that predicted the IAS scores primarily included connections of the prefrontal cortex with the cerebellum and limbic lobe, connections of the occipital lobe with the limbic lobe and insula lobe (Figure 3).

**Fig. 4.**
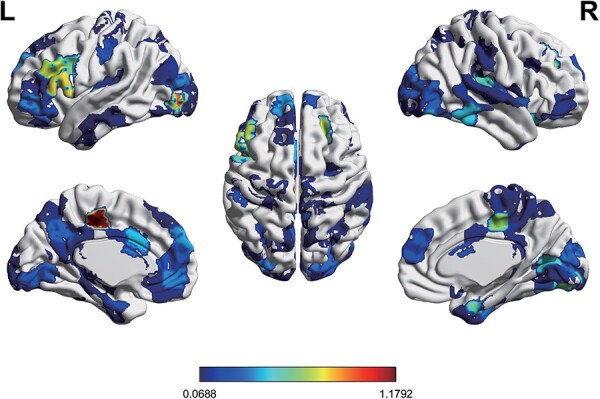
The node strength of the contributing network after controlling for head motion, age, gender and negative emotion scores. The correlation coefficients between the contributing network and the IAS scores were used as the weights of links, and then the node strength was computed by summing the absolute values of the correlation coefficients. Higher node strength represented a greater contribution to the prediction of IAS.

Additionally, the node strength was used to measure the importance of each node. The node strength was calculated as the sum of the link weights connected to that node. The link weights were defined as the correlation coefficients between the contributing network and the IAS scores. The sum of the absolute values of the correlation coefficients between all of the edges of the node in the contributing network and IAS scores was defined as the node strength (because all correlation coefficients have negative values). Regions with higher node strength were mainly located in median cingulate cortex (MCC), lateral occipital cortex (LOC), insula and ventrolateral prefrontal cortex (VLPFC) (Figure 4 and Table 2).

**Table 2.** Ten nodes with the greatest node strength selected by the prediction model

No.	Node strength	Node name	Network	L/R	Lobe	MNI coordinates
1	1.18	MCC	Somato-motor	L	Limbic	−7.8, −22.4,46
2	0.95	LOC	Visual	L	Occipital	−36, −84.2, −3.9
3	0.90	Insula	Subcortical	L	Insula	−37.7, −12.9, −1.4
4	0.79	VLPFC	Frontal-parietal	L	Prefrontal	−53.1,18.4,10.6
5	0.79	Lobule Crus2	n/a	R	Cerebellum	41.9,−64,−49.2
6	0.74	Inferior frontal gyrus	Frontal-parietal	L	Prefrontal	−46.1,28.2,26.8
7	0.72	Middle frontal gyrus	Default mode	R	Prefrontal	23.9,30.7,36.4
8	0.66	MCPC	Somato-motor	R	Limbic	7.8,−23.1,44.9
9	0.64	Lingual gyrus	Visual	R	Occipital	17.9,−83.4,−11.3
10	0.61	Inferior frontal gyrus	Frontal-parietal	L	Prefrontal	−46.2,7.9,28.6

Abbreviations: L, left hemisphere; R, right hemisphere; MNI, Montreal Neurological Institute; n/a, not available.

### Validation with different cross-validation schemes

**Fig. 5.**
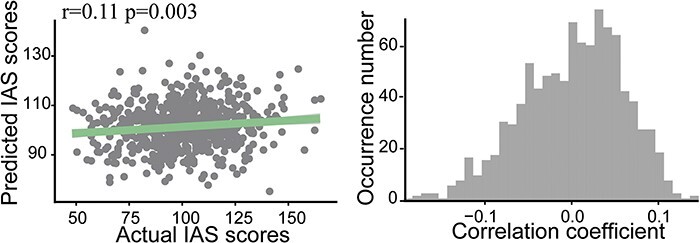
The validation performance of the predictive model after controlling for head motion, age, gender and negative emotion scores. The left graph shows the correlation between the actual and predicted IAS scores. The right graph depicts the permutation distribution of the correlation coefficient (*r*) for the prediction analysis.

To validate the performance of the predictive model, the 10-fold cross-validation approach was used. The significant results were replicated. The IAS scores in the independent subjects were predicted by the functional connectivity in the negative network (correlation between the actual and predicted scores: *r*_partial_ = 0.11, *P*_perm_ = 0.01, permutation test, Figure 5). Similarly, the functional connectivity in the positive network could not reliably predict the IAS scores (correlation between the actual and predicted scores: *r* = −0.02, *P* > 0.05).

### External generalizability

**Fig. 6.**
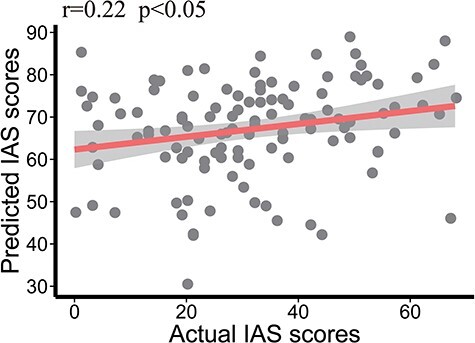
The correlation between the actual and predicted IAS scores in external validation.

Subsequently, we proceeded to evaluate the generalizability of the networks that predicted IAS in Dataset 1, which consisted of 677 participants, to an independent dataset of 115 participants (Dataset 2). Regression models were applied to the external generalizability dataset to determine whether the predicted IAS scores generated by models utilizing the set of edges identified in Dataset 1 exhibited a significant relationship with the observed IAS scores within the generalizability dataset. Notably, the findings revealed a noteworthy prediction of IAS for the negative network (*r* = 0.22, *P* = 0.017, as depicted in Figure 6). These outcomes indicate that the set of edges identified within the negative network possesses robustness in predicting individual variations in IAS.

### Comparing the sets of functional connections related to IAS to those related to AUDS

Finally, to identify unique prediction network specific to IAS, we sought to examine the differentiation between the sets of functional connections linked to IAS and AUDS.

To compare these networks, we constructed the neural predictive models of AUDS. After controlling for head motion, age, gender, negative emotion scores, negative networks still significantly predicted AUDS scores (correlation between the actual and predicted scores: *r*_partial_ = 0.16, *P*_partial_ = 0.03). In all iterations of LOOCV, the number of predictive model edges varied from 977 to 1675, with 411 edges appearing in each iteration defined as the contributing network (Supplementary Figure S1). The node strength of the contributing network was showed in Supplementary Figure S2.

**Fig. 7.**
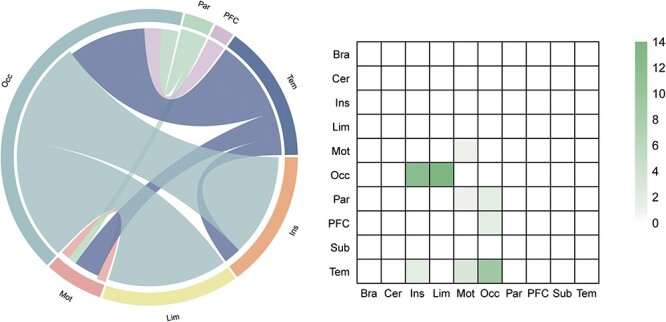
The unique contributing network that predicted the IAS scores compared to prediction networks of AUDS. The left graph shows the connections within and between each macroscale regions. The right graph depicts the connections plotted as number of edges within and between each pair of macroscale regions. PFC, prefrontal cortex; Mot, motor lobe; Ins, insular lobe; Par, parietal lobe; Tem, temporal lobe; Occ, occipital lobe; Lim, limbic lobe; Cer, cerebellum; Sub, subcortical lobe; Bsm, brainstem.

The findings reveal a minimal level of overlap between the networks. Among the 127 connections present in the negative IAS network, only two edges are shared with the negative AUDS network. This indicates that the set of functional connections predicting IAS largely differs from those predicting AUDS. Additionally, considering the macroscale regions, the unique contributing network that predicted IAS scores prominently consisted of connections between the occipital lobe and other lobes, such as the insula lobe, limbic lobe and temporal lobe (as shown in Figure 7).

## Discussion

With the popularity of the Internet, IAS has gradually emerged as a widespread public health problem threatening the physical and mental health of the population (Skopje *et al*., 2017). To date, no effort has been made to develop a model that can predict IAS at the individual level. Given the general consensus among researchers that each individual’s rsFC pattern is as unique and reliable as a fingerprint and underlies individual variation in personality characteristics and cognitive performance, brain characteristics provide promising candidates for predictive models that are important for the prevention and diagnosis of IAD (Braun *et al*., 2018). In this study, the CPM approach was used to build negative network based on whole-brain functional connectomes that predicts IAS at the individual level. CPM further indicated that the set of connections in the negative network related to IAS identified in one sample generalized (Dataset 1) to predict IAS in an independent sample (Dataset 2), demonstrating the replicability of this effect. Furthermore, our analysis revealed that the unique contributing network, which predicted IAS in contrast to the prediction networks of AUDS, prominently comprised connections involving the occipital lobe and other lobes, namely the insula lobe, limbic lobe and temporal lobe.

As previously mentioned, intrinsic functional connectivity spanning distributed networks can be used to predict individual IAS. Importantly, interindividual differences in IAS can be explained mainly by the functional connectivity of the prefrontal cortex with the cerebellum and limbic lobe, connections of the occipital lobe with the limbic lobe and insula lobe. The activity within these neural systems has been implicated in cognitive, affective, motor and visual components of IAS. Our findings indicate that these apparently disparate processes do not work in isolation but interact extensively to maintain IAS. Supporting evidence from the animal studies indicates that the addictive behavior has an irreversible impact on the brain chemistry and neural pathways (O’Brien and Gardner, 2005; Kupnicka *et al*., 2020).

This study demonstrated that the contributing prediction network of IAS included critical regions involved in cognitive processing of brain functioning, such as the MCC, LOC, insula and VLPFC. Prior studies have revealed that the MCC is involved in the anticipation and processing of rewards as well as reward-related decision-making (Liu *et al*., 2011). The addiction behavior involves long-term, persistent dysregulation of the activity in the brain reward systems mediating natural rewards and recruitment of brain stress (Koob, 2013; Ruisoto and Contador, 2019). Therefore, an impaired reward system function may be responsible for the development and maintenance of addictive behaviors. LOC, known as a high-level visual area, plays a crucial role in object perception and object size perception (Larsson and Heeger, 2006). Previous studies have additionally reported a decrease in the cortical thickness of the LOC in individuals with IGD (Wang *et al*., 2018). It has been postulated that individuals with IAS, who have been extensively engaged with the Internet, need to diligently attend to even subtle changes on their screens. Prolonged hypertension of visual attention can lead to impairments in visual functions (Dong *et al*., 2012a). Accordingly, alterations in the functional connectivity of this region may be associated with impairments in the attentional processing of visual information.

In addition to the MCC and LOC, the VLPFC and insula were mainly involved in the prediction of IAS. The VLPFC is related to decision-making that includes uncertainty or risk (McClure *et al*., 2004). The VLPFC interprets cognitive and motivational information and conducts inhibitory signaling of responses that must be cancelled or blocked to facilitate decision making, leading toward goal-directed behavior (Sakagami and Pan, 2007). The observed alterations in functional connectivity of the VLPFC in individuals with IAS may indicate inhibitory control or regulatory impairments that are potentially linked to addictive behaviors. This inhibitory dysfunction can be affected by environmental contingencies and cues (e.g. craving for the Internet) and consequently contributes to the maintenance of Internet abuse. The insula has been recognized as a key brain region responsible for integrating interoceptive states and self-awareness, leading to conscious emotional experiences. This integration is particularly crucial for decision-making processes involving risk and reward, as demonstrated in studies on drug craving and relapse (Naqvi *et al*., 2014). The observed alterations in the functional connectivity of the insula can potentially undermine the ability to effectively integrate information pertaining to risk and value during decision-making. Thus, these changes may impair the decision-making capacity related to assessing risks and rewards in individuals with IAS.

Finally, our results demonstrated that the set of functional connections that predict IAS are largely distinct from the set of functional connections that predict AUDS. Based on the macroscale regions, compared to prediction networks of AUDS, the unique contributing network that predicted the IAS scores primarily included connections of the occipital lobe with the other lobe, including insula lobe, limbic lobe and temporal lobe. The occipital lobe, known as the primary visual processing center in the brain, encompasses the major portion of the visual cortex. It is primarily responsible for visual functions (Kojima and Suzuki, 2010). Previous research has indicated the significant involvement of the occipital lobe in IAS (Dong *et al*., 2012b). Tasks related to Internet gaming have been found to activate the occipital gyrus, which constitutes the visual processing center (Liu *et al*., 2016). Notably, studies have reported decreased regional homogeneity in temporal, occipital and parietal brain regions among individuals with IAS, with these regions being associated with visual and auditory functions (Dong *et al*., 2012a). In our study, we found that the unique predictive network for IAS scores primarily involved connections between the occipital lobe and other brain lobes. This underscores the important and distinctive role played by the occipital lobe in predicting IAS when compared to the prediction networks for AUDS. Indeed, IAS has the problem of excessive use of electronic products, and the occipital lobe is more involved than AUDS.

Notably, our research is in part representative of progress in neuroscience and advances the application of machine-learning methods that use functional brain connections as feature values to build neuroimaging-based behavior prediction. The CPM is designed to uncover critical brain features that can be applied to improve the accuracy of diagnosis and the success of treatment for IAS in clinical practice (Yang *et al*., 2023). Within this framework, a growing number of studies have developed predictive models based on brain imaging features to distinguish those with IAD from healthy controls or to predict symptom severity. Therefore, the rsFC-based predictive model for IAS could be used to intervene in potentially at-risk populations in a timely manner and to generate effective therapeutic measures for IAD.

Nevertheless, there are limitations of the present study. First, while the main confounding factors such as age, sex, head movement and negative emotion were controlled in this study, other behaviors variably associated with IAS (e.g. objective time spent online) should be measured and controlled in future studies. Second, all measures of behavior are also limited by subjective self-reporting rather than objective measures of performance. Third, our predictive model was constructed with a healthy population, and future research should be performed so that the results can be generalized to patients with IAD by applying a machine-learning technique that can classify those with IAD and healthy individuals. Finally, heavy chronic alcohol use introduces its own neurotoxicity and effects on brain connectivity (Rao and Topiwala, 2020) that could suppress or interfere with detection of parallels in prediction of AUDS with prediction of IAS.

Despite these limitations, this study first demonstrated that the functional connectivity of distributed networks effectively predicts IAS at the individual level. Importantly, the brain regions of the prediction network involve cognitive, affective, motor and visual processes, and deficits in these processes are closely related to addictive behaviors. Furthermore, we found the important and unique role of the occipital lobe in predicting IAS. In a word, our research provides a deeper understanding of the neural mechanisms of IAS within novel frameworks and has potential clinical applications in the prevention and intervention of IAD.

## Supplementary Material

nsae007_Supp
