# Supplementary material for: Connectome-based predictive modeling of Internet addiction symptomatology
Source: Soc Cogn Affect Neurosci. 2024 Feb 9;19(1):nsae007. doi: 10.1093/scan/nsae007 (PMC10878364; doi:10.1093/scan/nsae007)
Supplement: nsae007_Supp [file nsae007_supp.zip › scan-23-081-File011.docx]

**Supplemental Materials for** **Connectome-based predictive modeling of Internet addiction symptomatology**

This document is organized as follows:

Table S1 The descriptive statistics of behavioral measurements in Dataset 2

Table S2 The Pearson correlation between Internet addiction symptomatology and negative emotion

Figure S1 Functional connections predicting the alcohol use disorder symptomatology

Figure S2 The node strength of the contributing network related to alcohol use disorder symptomatology

| outcomes | N | Mean | SD | Range |
| --- | --- | --- | --- | --- |
| AUDS | 205 | 2.31 | 3,29 | 0-27 |
| Depression | 195 | 5.95 | 6.19 | 0-29 |
| State Anxiety | 195 | 39.13 | 10.14 | 20-72 |
| Perceived Stress | 198 | 23.71 | 6.93 | 9-42 |
| Negative Affect | 195 | 16.80 | 6.17 | 9-36 |
| Loneliness | 198 | 39.39 | 8.37 | 20-64 |
| IAS | 115 | 31.16 | 16.76 | 0-68 |

Table S1 The descriptive statistics of behavioral measurements in Dataset 2

Table S2 The Pearson correlation between Internet addiction symptomatology and negative emotion

|  | IAS | state anxiety | depression | perceived stress | negative affect | loneliness |
| --- | --- | --- | --- | --- | --- | --- |
| IAS |  |  |  |  |  |  |
| state anxiety | 0.294*** |  |  |  |  |  |
| depression | 0.243*** | 0.419*** |  |  |  |  |
| perceived stress | 0.275*** | 0.497*** | 0.320*** |  |  |  |
| negative affect | 0.362*** | 0.535*** | 0.377*** | 0.633*** |  |  |
| loneliness | 0.369*** | 0.482*** | 0.401*** | 0.479*** | 0.496*** |  |

*** p < 0.001


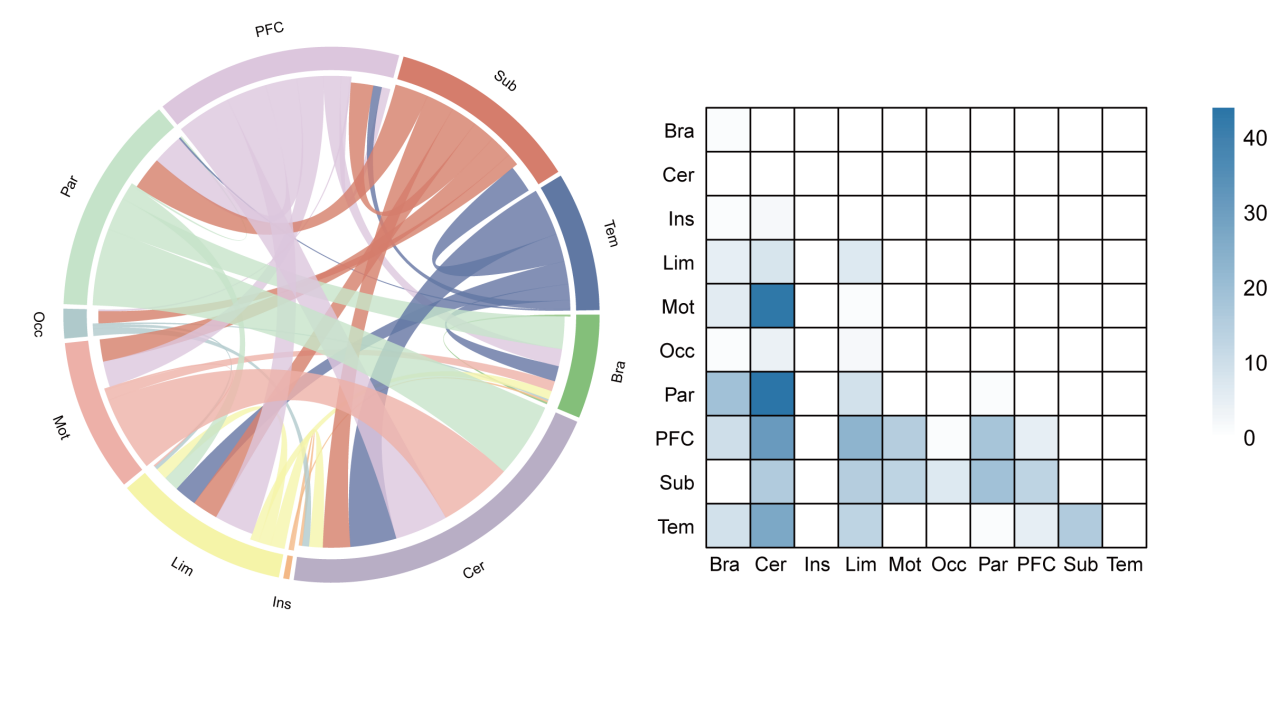


Figure S1 Functional connections predicting the alcohol use disorder symptomatology. The left graph shows the connections within and between each macroscale regions. The right graph depicts the connections plotted as number of edges within and between each pair of macroscale regions. PFC, prefrontal cortex; Mot, motor lobe; Ins, insular lobe; Par, parietal lobe; Tem, temporal lobe; Occ, occipital lobe; Lim, limbic lobe; Cer, cerebellum; Sub, subcortical lobe; Bsm, brainstem.


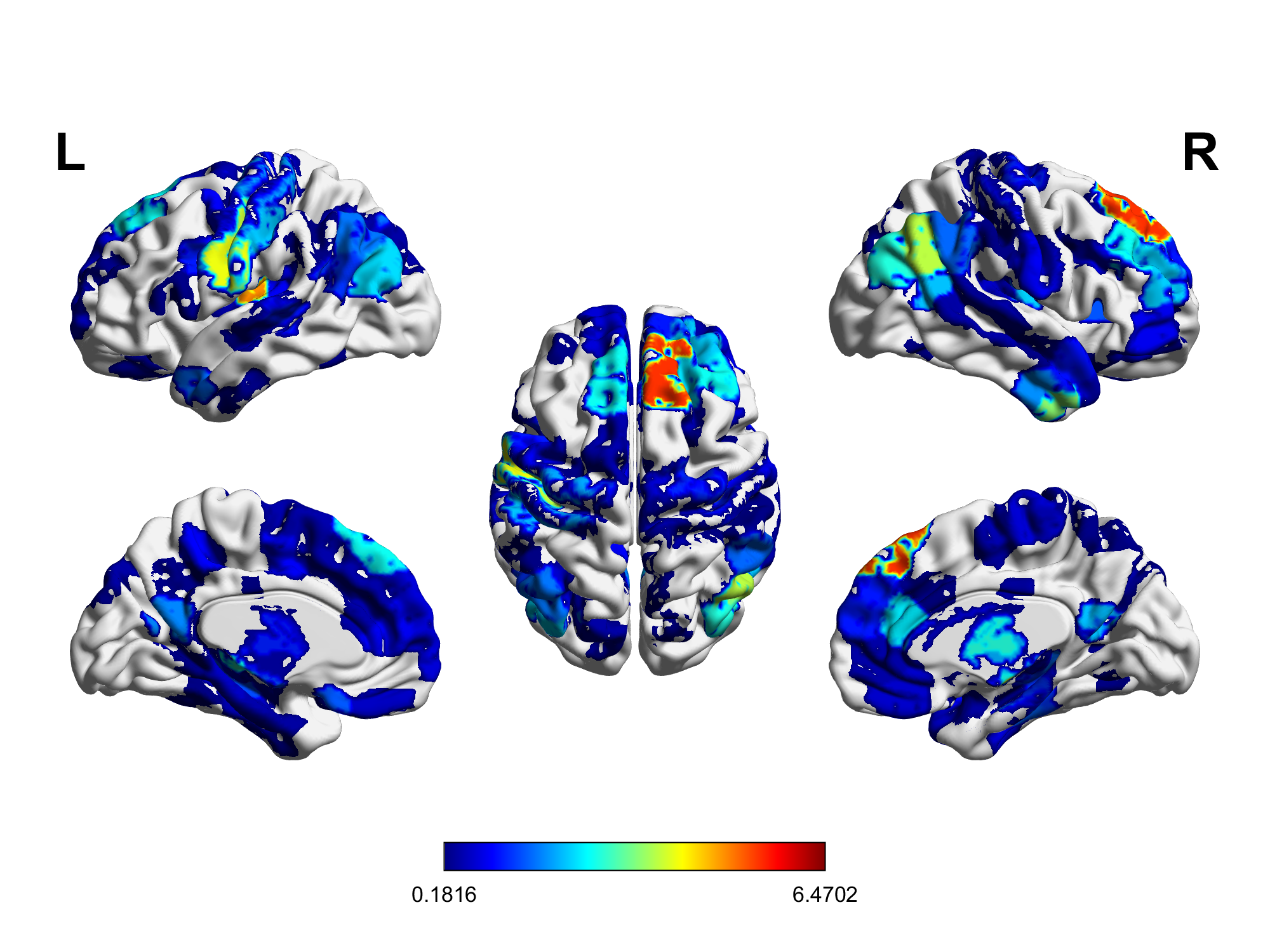


Figure S2 The node strength of the contributing network related to alcohol use disorder symptomatology. The correlation coefficients between the contributing network and the alcohol addiction were used as the weights of links, and then the node strength was computed by summing the absolute values of the correlation coefficients. Higher node strength represented a greater contribution to the prediction of alcohol addiction.
